# OA27 Beyond Juvenile Idiopathic Arthritis: Lessons from an Adolescent with an Ultra-Rare Skeletal Disorder.

**DOI:** 10.1093/rap/rkag113.027

**Published:** 2026-11-03

**Authors:** Rosanna Massie, Marcus Sim, Anne-Marie McMahon, Samantha Leach, Catherine Dunbar

**Affiliations:** Sheffield Children’s NHS Foundation Trust, Sheffield, United Kingdom; Sheffield Children’s NHS Foundation Trust, Sheffield, United Kingdom; Sheffield Children’s NHS Foundation Trust, Sheffield, United Kingdom; Sheffield Children’s NHS Foundation Trust, Sheffield, United Kingdom; Sheffield Children’s NHS Foundation Trust, Sheffield, United Kingdom

## Abstract

**Introduction:**

A young person’s diagnostic journey across three countries with different healthcare systems will be presented. Initially diagnosed as juvenile idiopathic arthritis, persistent erosions and osteolysis led to treatment escalation followed by a search for an alternative diagnosis. Genetic testing ultimately revealed a diagnosis of multicentric carpotarsal osteolysis, a skeletal disorder presenting in childhood with a clinical picture mimicking juvenile idiopathic arthritis. Navigating diagnostic uncertainty in the face of progressive joint damage and loss of function, as well as the uncertain role of immunosuppression and limited evidence base for appropriate therapy intervention highlights the challenge of managing this ultra-rare condition.

**Case description:**

The case focuses on a thirteen-year-old boy with a diagnosis of multicentric carpotarsal osteolysis. He has no significant medical history other than attention deficit hyperactivity disorder. We were made aware of him following recent relocation from Bermuda to the UK in May 2025, his condition having previously been managed by local health services and Boston Children’s Hospital.

At age three, his parents initially noticed thumb changes. Over the years, this progressed to tingling in his hands and feet, with poor healing from sport-related wrist and foot fractures. Abnormalities identified on radiographs following the delayed healing of these fractures initiated further investigation that led to a diagnosis of juvenile idiopathic arthritis.

Methotrexate and adalimumab were commenced together, with adalimumab later increasing to weekly injections. When symptoms remained unresponsive, genetic testing was pursued. This identified a heterozygous pathogenic mutation for MAFB c.161C>T, (p.Ser54Leu), consistent with a diagnosis of multicentric carpotarsal osteolysis. Methotrexate and adalimumab were stopped 6 months from initiation with no worsening of symptoms.

Multi-disciplinary support was provided by Bermuda/Boston, including physiotherapy, occupational therapy, podiatry and psychology involvement. Unfortunately, the child continues to face progressive distal joint pain and bone deformity of the wrists and feet, significantly impacting daily activity and broader wellbeing.

On relocation to the UK, care was transferred to the rheumatology multi-disciplinary team at Sheffield Children’s Hospital with input from metabolic bone and paediatric nephrology.

Magnetic resonance imaging of more symptomatic joints (left wrist and left foot/toes) is being planned to assess if there is significant local inflammation that may be amenable to targeted intraarticular corticosteroid injection. A dietetic referral has also been made for low body-mass index <0.4th centile.

Therapy management is focused on maintaining joint range of motion, preventing further muscle contractures and supporting global strengthening and cardiovascular fitness.

**Discussion:**

Multicentric carpotarsal osteolysis (MCTO) is a rare condition with limited literature available on the specific medical and therapeutic recommendations for ongoing management.

Some case reports describe some improvement in symptoms and function with immunosuppressive agents, although no impact on radiographic progression. The unclear pathogenesis and uncertain role for immunosuppression raises further questions such as the role of systemic therapies or corticosteroids in treating localised joint inflammation to improve symptoms and function. The young person previously received corticosteroids, methotrexate, and adalimumab, albeit for 6 months, with minimal improvement in symptoms.

Anti-resorptive agents (bisphosphonates or denosumab) have also been explored, although do not appear to be disease-modifying, with the metabolic bone teams in Sheffield and Boston feeling any benefit would be outweighed by the possible adverse effects (e.g. calcium dysregulation following discontinuation). Although there are some reports of low mineral bone density in some cases of MCTO, this has not been the case for the young person.

The case also highlights the role for genetic testing, especially in cases of unusually progressive or refractory juvenile arthritis. Identification of mimics, whether inflammatory or non-inflammatory, is critical to the appropriate use of immunosuppression. However, there are challenges with the increasing identification of rare genetic variants, especially when targeted therapies are limited. Navigating a rare and complex diagnosis with the family where management is uncertain requires specialist input from a clinician familiar with managing rare diseases. Personalising this care requires close multi-disciplinary working as well as utilising expert patient experiences in these evidence-poor situations.

Non-pharmacological management including supportive rehabilitative physiotherapy and occupational therapy play a vital role, although specific recommendations do not exist due to the rarity of the diagnosis. Universal advice around type or intensity of physical activity do not exist. Balancing joint-protective recommendations without unnecessarily limiting the young person’s options remains a challenge.

**Key learning points:**

• The main learning point is the challenges of multidisciplinary management of an ultra-rare condition where evidence for effective intervention is either very limited or non-existent.

• The case also highlights the importance of coordinated multi-disciplinary input when managing rare complex conditions to ensure holistic care of the young person, with additional considerations for the unique psychosocial and developmental needs of adolescents

• A further point for discussion is the management of patient/parental expectation and anxiety following a complex journey to diagnosis in another country, and varied opinions on best management from different healthcare services across three countries. To give an example, the family have previously been advised against physical activity, however limiting participation has had a significant impact on mental health and physical conditioning. With minimal literature available there is significant challenge to advising on the balance of quality of life and mental wellbeing without risking the possibility of exacerbating disease progression. In pursuit of clarity, support from the trust library has been ascertained to conduct a thorough review of the published literature.

• The case also highlights the role of genetics in unusual presentations of inflammatory arthritis.

• Presenting at the conference will provide an excellent opportunity for open discussion with other rheumatology centres about this ultra-rare condition. It would be helpful to know if this condition is familiar to other paediatric centres specifically and how multidisciplinary management has been approached.

• Presentation will also raise awareness of the need for research into this condition and clarify whether this is currently being undertaken in the UK or is something for the team at Sheffield Children’s Hospital to initiate, to inform future management guidelines.

